# Circadian nutritional behaviours and risk of cardiovascular disease in NutriNet-Santé

**DOI:** 10.1093/eurpub/ckac129.551

**Published:** 2022-10-25

**Authors:** B Srour, A Palomar-Cros, VA Andreeva, LK Fezeu, C Julia, A Bellicha, E Kesse-Guyot, D Romaguera, M Kogevinas, M Touvier

**Affiliations:** 1 EREN, Inserm, Inrae, Cnam, USPN, INSERM, Bobigny, France; 2 Barcelona Institute for Global Health, ISGlobal, Barcelona, Spain; 3 Avicenne hospital, Public Health Department, Bobigny, France; 4 IdISBa, Palma de Mallorca, Spain; 5 CIBEROBN, Madrid, Spain; 6 IMIM, Barcelona, Spain; 7 CIBERESP, Institute of Health Carlos III, Madrid, Spain; 8 Equal contributions

## Abstract

Meal timings and daily night-time fasting periods can synchronise the circadian system, which regulates the cardiovascular system. The present study aims to evaluate the prospective associations between circadian nutritional behaviours, defined by meal timing and frequency, and the risk of cardiovascular diseases. We used data from 103,389 adults (79% females) in the French NutriNet-Santé study, 2009-2021. Circadian nutritional behaviours were assessed using repeated 24h food records during the first two years of follow-up. We examined the associations between circadian eating behaviours and risk of cardiovascular, coronary heart and cerebrovascular diseases by multivariable Cox proportional hazard models. During a median follow-up of 7.2 years, 2036 incident cardiovascular diseases were diagnosed. A later first meal of the day was associated with a higher risk of cardiovascular diseases (HR per hour increase = 1.06, 95% CI 1.01 - 1.12). A later last meal of the day was associated with a higher risk of cerebrovascular diseases (HR per hour increase = 1.08, 95% CI 1.01 - 1.15). Among women, a later last meal was also associated with a higher risk of cardiovascular disease (HR per hour increase = 1.08, 95% CI 1.01 - 1.15). We found no evidence for an association between night-time fasting duration nor meal frequency, with risk of cardiovascular diseases. This study suggests that the habit of eating a later first meal, and a later last meal (in women) could be associated with a higher risk of developing circulatory diseases. These results need to be confirmed in other largescale studies before they can be transferable to clinical practice.

**Key messages:**

• Beyond nutritional quality of meals, meal timing could also be a risk factor for cardiovascular disease.

• If confirmed in other largescale studies, early breakfast and dinner could be considered in preventive strategies of cardiovascular diseases.

